# Molecular Cloning and Functional Characterization of the *Lycopene ε-Cyclase* Gene via Virus-Induced Gene Silencing and Its Expression Pattern in *Nicotiana tabacum*

**DOI:** 10.3390/ijms150814766

**Published:** 2014-08-22

**Authors:** Yanmei Shi, Ran Wang, Zhaopeng Luo, Lifeng Jin, Pingping Liu, Qiansi Chen, Zefeng Li, Feng Li, Chunyang Wei, Mingzhu Wu, Pan Wei, He Xie, Lingbo Qu, Fucheng Lin, Jun Yang

**Affiliations:** 1 Department of Chemistry, Zhengzhou University, Zhengzhou 450001, China; E-Mail: symzgh@163.com; 2 National Tobacco Gene Research Center, Zhengzhou Tobacco Research Institute, Zhengzhou 450001, China; E-Mails: wangranljj2010@163.com (R.W.); zhiwubingdu@sohu.com (Z.L.); jin_lf@126.com (L.J.); spesge@163.com (P.L.); nanzhichan@gmail.com (Q.C.); ibi.zefeng@gmail.com (Z.L.); likite2002@163.com (F.L.); ztrisam@126.com (C.W.); mingzhuwus@126.com (M.W.); weipan83@126.com (P.W.); fuchenglin@zju.edu.cn (F.L.); 3 Molecular Breeding Group, Yunnan Academy of Tobacco Agricultural Sciences, Kunming 650031, China; E-Mail: xieh@tobacco.gov.cn; 4 College of Chemistry and Chemical Engineering, Henan University of Technology, Zhengzhou 450001, China; E-Mail: qulingbo@zzu.edu.cn

**Keywords:** lycopene ε-cyclase, functional characterization, virus-induced gene silencing, *Nicotiana tabacum*, carotenoid biosynthesis

## Abstract

Lycopene ε-cyclase (ε-LCY) is a key enzyme that catalyzes the synthesis of α-branch carotenoids through the cyclization of lycopene. Two cDNA molecules encoding *ε-LCY* (designated *Ntε-LCY1* and *Ntε-LCY2*) were cloned from *Nicotiana tabacum*. *Ntε-LCY1* and *Ntε-LCY2* are encoded by two distinct genes with different evolutionary origins, one originating from the tobacco progenitor, *Nicotiana sylvestris*, and the other originating from *Nicotiana tomentosiformis*. The two coding regions are 97% identical at the nucleotide level and 95% identical at the amino acid level. Transcripts of *Ntε-LCY* were detectable in both vegetative and reproductive organs, with a relatively higher level of expression in leaves than in other tissues. Subcellular localization experiments using an Ntε-LCY1-GFP fusion protein demonstrated that mature Ntε-LCY1 protein is localized within the chloroplast in Bright Yellow 2 suspension cells. Under low-temperature and low-irradiation stress, *Ntε-LCY* transcript levels substantially increased relative to control plants. Tobacco rattle virus (TRV)-mediated silencing of *ε-LCY* in *Nicotiana benthamiana* resulted in an increase of β-branch carotenoids and a reduction in the levels of α-branch carotenoids. Meanwhile, transcripts of related genes in the carotenoid biosynthetic pathway observably increased, with the exception of *β-OHase* in the *TRV-ε-lcy* line. Suppression of *ε-LCY* expression was also found to alleviate photoinhibition of Potosystem II in virus-induced gene silencing (VIGS) plants under low-temperature and low-irradiation stress. Our results provide insight into the regulatory role of *ε-LCY* in plant carotenoid biosynthesis and suggest a role for *ε-LCY* in positively modulating low temperature stress responses.

## 1. Introduction

Leaves of flue-cured tobacco cultivars are the main raw materials for the tobacco industry in East Asia. Carotenoids are not only important for the development of leaves of flue-cured tobacco plants, but also contribute to the quality of mature tobacco leaves, in that many flavor components come from the cracking of carotenoids. Carotenoids are isoprenoid pigments, often highly colored, that are synthesized in plants, as well as in some bacteria, fungi and algae. In higher plants, carotenoids play crucial roles in photosynthesis, photoprotection [1] and the production of carotenoid-derived phytohormones, including abscisic acid (ABA) [2,3] and strigolactone [4,5,6]. In humans, carotenoids are a necessary dietary component for proper health, being an essential source of retinoids and vitamin A, the deficiency of which leads to xerophthalmia, blindness and premature death [7,8,9,10]. In addition, carotenoids have been shown to promote human health through their antioxidant activity, immunostimulation and photoprotection functions [11,12]. Therefore, increasing the content of carotenoids in plants is of great interest for nutritional enhancement.

Carotenoids are products of the isoprenoid biosynthetic pathway. The genes encoding the enzymes of the main steps of the carotenoid biosynthetic pathway have been extensively studied in model plants [13]. Carotenoid biosynthesis begins with the formation of phytoene from geranylgeranyl diphosphate catalyzed by phytoene synthase (PSY). Three enzymes, including phytoene desaturase (PDS), ζ-carotene desaturase (ZDS) and carotenoid isomerase (CRTISO), convert phytoene to lycopene via phytofluene and ζ-carotene. Carotenoid biosynthesis bifurcates after lycopene to produce α- and β-carotenoids through the action of two lycopene cyclases: ε-LCY and lycopene β-cyclase (β-LCY). One route leads to the formation of carotenoids with one β and one ε end ring, such as α-carotene and lutein. Lutein is a major xanthophyll in the light-harvesting system of most plants. The alternative pathway leads to β-carotene and its, derivatives, zeaxanthin, violaxanthin and neoxanthin. Violaxanthin and neoxanthin are the precursors for the synthesis of ABA. The relative activities of ε-LCY and β-LCY may determine the flow through the carotenoid pathways from lycopene to α- or β-carotene and their derivatives [14,15,16].

Several mutations to genes in the pathway, including *lut1* (*ε-hydroxylase*) [17], *lut2* (*ε-LCY*) [18,19], *lut5* (*β-hydroxylase*) [20], *ccr2* (*CRTISO*) [21] and *ccr1* (SDG8 chromatin regulatory mutant) [22], distinctively affected the ratio of α- and β-branch metabolites. Prior studies have indicated that *ε-LCY* activity is the critical step that coordinates carotenoid content in plants [14,23,24,25,26]. Natural variation of *ε-LCY* gene expression levels can explain 58% of the variation in the lutein and β-carotenoid content in maize [23]. Suppression of *ε-LCY* expression in *Arabidopsis* resulted in a marked reduction in the ratio of lutein to β-carotene [24]. Downregulation of *ε-LCY* transcript levels by RNAi enhanced carotenoid synthesis via the β-branch-specific pathway, and reduced the lutein content in sweet potato [25]. The β-carotenoid content increased significantly through the tuber-specific silencing of *ε-LCY* expression in potato, but this was not accompanied by a concomitant decrease in lutein content [26]. In seeds of *Brassica napus*, the down-regulation of *ε-LCY* expression by RNAi resulted in higher levels of total carotenoids, with increased β-carotene, zeaxanthin, violaxanthin and, unexpectedly, lutein [14]. These studies suggested that different genetic regulatory mechanisms might be operating on *ε-LCY* in different plants. Tobacco is not only an important economic crop, but also an excellent model plant. Tobacco has played a pioneering role in plant research, laying the groundwork for modern agricultural biotechnology. Therefore, developing the research of *ε-LCY* gene in tobacco is of great significance.

Analysis of gene function in some higher plants is often hampered by the fact that stable genetic transformation to down- or up-regulate gene expression is both laborious and slow. Virus-induced gene silencing (VIGS) has proven to be a powerful tool for the rapid analysis of gene function. It is possible to target most genes and downregulate the mRNA levels in a sequence-specific manner [27]. *Nicotiana benthamiana* (*N. benthamiana*) is by far the best-studied host for VIGS, and the VIGS response is generally stronger and more persistent in *N. benthamiana* than in other plants [28]. Several plant viruses, such as potato virus X (PVX) [29], tobacco mosaic virus (TMV) [30] and tobacco rattle virus (TRV) [31,32] have been adapted for use as VIGS vectors to downregulate a given endogenous plant target gene after inoculation of the plant with the VIGS vector. TRV is particularly widely used for gene functional characterization in many plant species [31,32,33,34], as TRV infection causes relatively mild disease symptoms and often induces intense and uniform silencing phenotypes. Moreover, genes expressed in floral organs and even pollen can be silenced with TRV-mediated VIGS methods.

In this study, we isolated and characterized two lycopene ε-cyclase genes (*Ntε-LCY1* and *Ntε-LCY2*) from *Nicotiana tabacum* (*N*. *tabacum*) and used TRV-mediated VIGS technology to analyze their functions in *N. benthamiana*. We found that *ε-LCY* gene silencing was accompanied by changes in carotenoid composition. We also found that suppression of *ε-LCY* expression affected the sensitivity of photosystem (II) to low-temperature and low-irradiance stress.

## 2. Results

### 2.1. Isolation and Characterization of Ntε-LCY Genes

**Figure 1:**
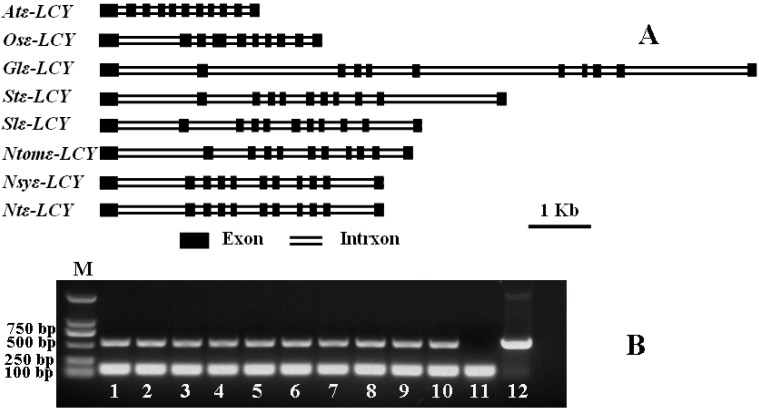
(**A**) Comparisons of the exon (box) and intron (double line) patterns of the *ε-LCY* genes from *Arabidopsis* (*Atε-LCY*), *Oryza sativa* (*Osε-LCY*), *Glycine max* (*Glε-LCY*), *Solanum tuberosum* (*Stε-LCY*), *Solanum lycopersicum* (*Slε-LCY*), *Nicotiana tomentosiformis* (*Ntomε-LCY*), *Nicotiana sylvestris* (*Nsyε-LCY*) and *Nicotiana tabacum* (*Ntε-LCY1*); (**B**) PCR amplification fragment of *ε-LCY* used IF primers from genomic DNA of *N. tabacum*, *N. sylvestris* and *N. tomentosiformis*.

An mRNA sequence designated *Ntε-LCY1* was cloned from a tobacco leaf cDNA library using PCR and oligonucleotide primers based on the LYCe (HQ993098.1) sequences available in NCBI databases. Comparison of *Ntε-LCY1* to other known *ε-LCY*s revealed protein sequence identities ranging from 90.9% for *Solanum tuberosum* to 72.6% for *Arabidopsis thaliana* (Supplementary File 1). Based on the high degree of similarity between *Ntε-LCY1* and the other plant *ε-LCYs* that we examined, we expected that the tobacco *Ntε-LCY1* may have similar biological functions as that of other plants. Subsequently, we used a similar approach to clone the full-length *Ntε-LCY1* gene from genomic DNA of *N*. *tabacum*. As shown in Figure 1A, the complete gene sequence extends precisely 4356 base pairs, with 11 exons and 10 introns. The coding sequences and full-length gene sequences of *ε-LCY* were cloned into the tobacco progenitor *Nicotiana sylvestris* (*N*. *sylvestris*) and *Nicotiana tomentosiformis* (*N*. *tomentosiformis*); the gene structures are shown in Figure 1A. To investigate the copy number of *Ntε-LCY* genes in tobacco, we compared the structure of *Ntε-LCY1* with that of the tobacco progenitor and other plants, designed a pair of intron flanking (IF) primers that were labeled by FAM spanning intron II and used these to amplify additional *ε-LCY* genes in tobacco. Figure 1B shows that there were two bands in ten varieties of *N. tabacum*, but only one band in the tobacco progenitors, *N. Sylvestris* and *N. tomentosiformis*. Sequencing results showed that the two bands in *N. tabacum* represented the *ε-LCY* genes of *N*. *sylvestris* and *N. tomentosiformis.* The products of PCR amplification in *N. tabacum* could be easily resolved by capillary electrophoresis, a method that can separate two DNA fragments if they differ in length base as few as one base pair (Supplementary File 2). Based on the electrophoresis and sequencing results, one fragment corresponded to *Ntε-LCY1* and the other corresponded to a second *Ntε-LCY* gene, henceforth referred to as *Ntε-LCY2*. We used intron length polymorphism information to deduce that there might be two copies of the *ε-LCY* gene in modern tobacco cultivars. Using the same strategy, one *ε-LCY* fragment was found in *N. sylvestris* and another was found in *N. tomentosiformis* (Supplementary File 3). The cDNA designated as *Ntε-LCY2* was subsequently cloned from a tobacco leaf cDNA library after sequencing of amounts of PCR products randomly. Sequencing analysis showed that the coding regions of *Ntε-LCY1* and *Ntε-LCY2* were highly similar to each other, with 97% identity at the nucleotide level and 95% identity at the amino acid level. The sequence of *Ntε-LCY2* was identical to that of HQ993098.1. Both *Ntε-LCY1* and *Ntε-LCY2* contained a single open reading frame of 1575 base pairs in length that encoded a protein of 524 amino acids.

**Figure 2:**
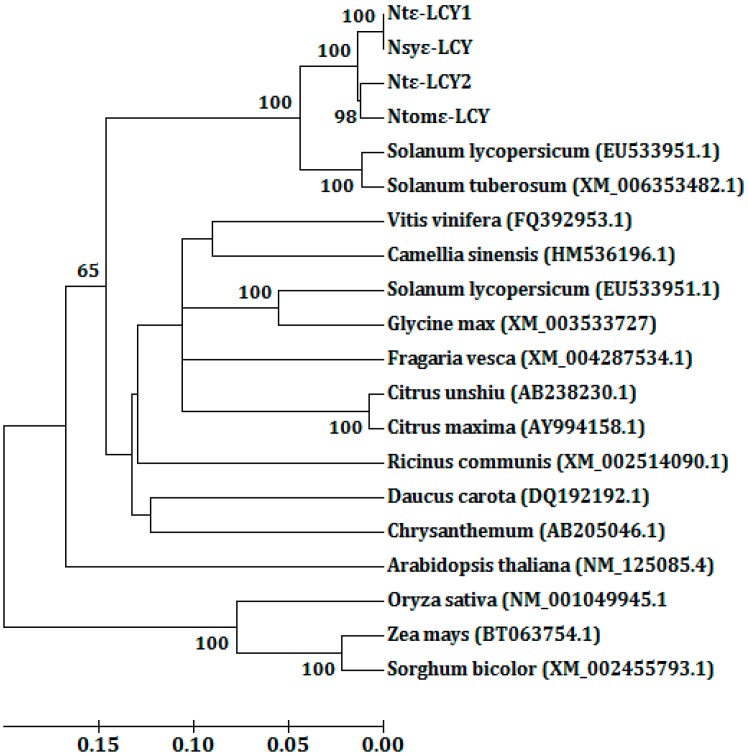
Phylogenetic analysis of the *ε-LCY* genes. The *ε-LCY* sequences from *N. tabacum* (*Ntε-LCY*), *N. sylvestris* (*Nsyε-LCY*), *N. tomentosiformis* (*Ntomε-LCY*) and other plants were used for this analysis. The tree shown was constructed using the neighbor joining method based on an alignment of the nucleotide sequences of *ε-LCY* genes. The bootstrap values were each estimated using 1000 replications.

The relationships between *Ntε-LCY1*, *Ntε-LCY2* and other plant *ε-LCYs* were further investigated by generating a phylogenetic tree (Figure 2). This analysis was conducted in MEGA5 using the UPGMA method [35]. For genetic distance analysis, bootstrap support was estimated using 1000 replicates. According to the phylogenetic tree, *Ntε-LCY1* was grouped with *N. sylvestris*, while *Ntε-LCY2* was more closely related to the sequence of *N. tomentosiformis*. Further, the identity between *Ntε-LCY1* and *Nsyε-LCY* was 99.5% at the nucleotide level, while the identity between *Ntε-LCY2* and *Ntomε-LCY* was 98.2% (Supplementary File 4).

To obtain evolutionary insights, we calculated the *K*s values between *Ntε-LCY1* and *Nsyε-LCY* and the *K*s values between *Ntε-LCY2* and *Ntomε-LCY*. The value of *K*s for *Ntε-LCY1* and *Nsyε-LCY* was 0.004, while that for *Ntε-LCY2* and *Ntomε-LCY* was 0.072. The rate of evolution for *Ntε-LCY1* and *Nsyε-LCY* was estimated to be 1.4 × 10^−8^ substitutions per site per year, whereas that for *Ntε-LCY2* and *Ntomε-LCY* it was 1.8 × 10^−7^ substitutions per site per year, based on the formation time (0.2 MYA) of *N. tabacum* [36], suggesting that *Ntε-LCY2* has evolved much faster than *Ntε-LCY1*. To further investigate the selective pressure exerted on *Ntε-LCY1* and *Ntε-LCY2*, the nonsynonymous/synonymous substitution rate ratios (*ω* = *K*a/*K*s) were calculated. The ratio for *Ntε-LCY1* and *Nsyε-LCY* was 1.09, which is indicative of relatively neutral selection on *Ntε-LCY1*. The ratio for *Ntε-LCY2* and *Ntomε-LCY* was 0.046, which indicated that *Ntε-LCY2* might have undergone purifying selection.

### 2.2. Subcellular Localization of Ntε-LCY1 Mature Protein

**Figure 3:**
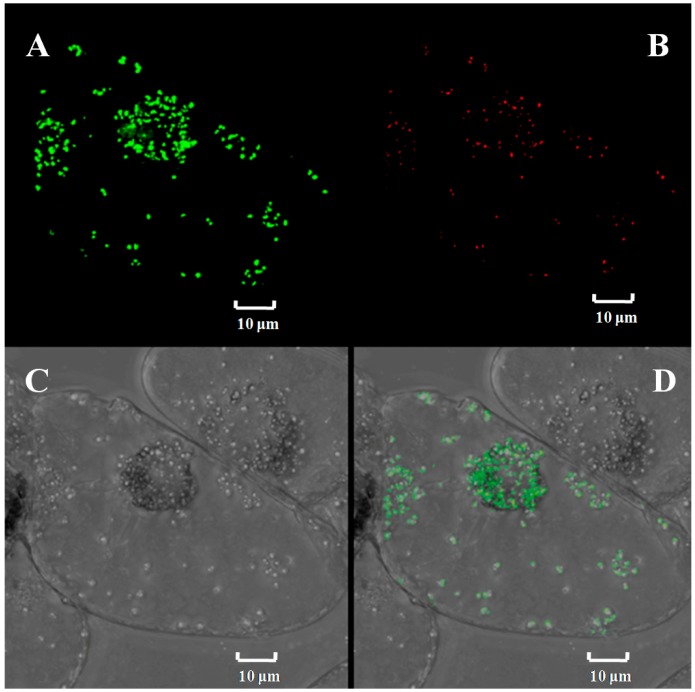
Subcellular localization of *Ntε-LCY*. The transient expression of Ntε-LCY-GFP fusion protein was observed in BY-2 cells using confocal microscopy. (**A**) Fluorescence of the fusion protein (green); (**B**) autofluorescence of chlorophyll (red); (**C**) bright field; (**D**) merged images.

Analysis of the protein encoded by *Ntε-LCY1* using the WoLF PSORT system (developed at Simon Fraser University, a program capable of predicting protein subcellular localization) suggested that Ntε-LCY1 was most probably localized in chloroplasts. To verify this prediction, we expressed an Ntε-LCY1-GFP fusion protein transiently in BY-2 cells and determined its localization according to the patterns of GFP fluorescence. Vectors encoding the Ntε-LCY1-GFP fusion protein were transformed into BY-2 cells by particle bombardment, and confocal microscopy was used to identify the location of Ntε-LCY1-GFP fusion protein. As is shown in Figure 3, the pattern of Ntε-LCY1-GFP fluorescence exactly coincided with that of the red auto-fluorescence of chloroplasts. Therefore, it could be concluded that Ntε-LCY1 is localized exclusively in chloroplasts.

### 2.3. Transcriptional Expression Patterns of Ntε-LCY

**Figure 4:**
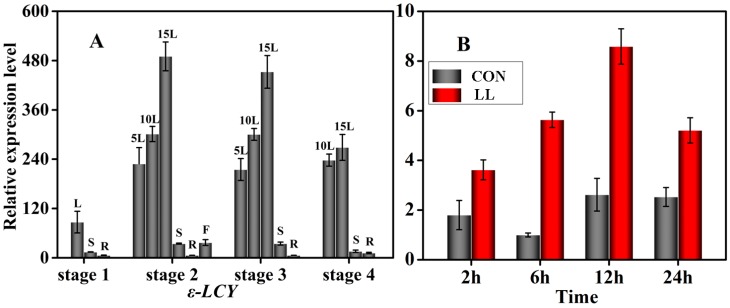
Gene expression pattern analysis of *Ntε-LCY*. (**A**) Spatio-temporal expression of *Ntε-LCY* in tobacco leaf (L), 5th leaf (5L), 10th leaf (10L), 15th leaf (15L), stem (S), root (R) and flower (F); (**B**) relative transcript levels of *Ntε-LCY* under conditions of normal (CON) and low temperature and low light (LL). The height of each bar represents the mean of three measurements, and the error bars indicate the standard deviation.

qRT-PCR was used to evaluate the transcript expression level of *Ntε-LCY* (*Ntε-LCY1* and *Ntε-LCY2*) in different developmental stages (Stages 1–4: seedling restitution stage, flowering stage, topping stage and leaf maturity stage) and different tissues (leaf, stem, root and flower) in *Nicotiana tabacum*. As is shown in Figure 4A, the transcript level of *Ntε-LCY* was higher in leaves than in any other organ at each stage and was higher in young leaves than in old leaves (transcript levels, 15L > 10L > 5L). Roots were the organ with the lowest *Ntε-LCY* transcript levels. Moreover, the transcript level of *Ntε-LCY* increased significantly from the seedling restitution stage to the flowering stage and then went through an obvious reduction by the leaf maturity stage. The high transcript levels in leaves were consistent with localization the of Ntε-LCY protein in chloroplasts. This transcriptional expression pattern was reproducibly obtained in multiple independent experiments.

The effects of low-temperature and low-irradiance stress on the expression of *Ntε-LCY* were examined in a time series from 2 to 24 h of stress treatment; the results of this analysis are illustrated in Figure 4B. The relative expression of *Ntε-LCY* increased obviously after 2 h under stress, and the highest expression levels were achieved between 6 and 12 h. When the stress time exceeded 24 h, the relative expression levels of *Ntε-LCY* decreased. These results suggested that low-temperature and low-irradiance stress can induce the expression of *Ntε-LCY*. The decrease in expression observed at the time point may have resulted from the effects of sustained stress on the overall vitality of the plants.

### 2.4. Manipulation of ε-LCY Expression Levels in Nicotiana Benthamiana and Its Effects on Carotenoid Content

**Figure 5:**
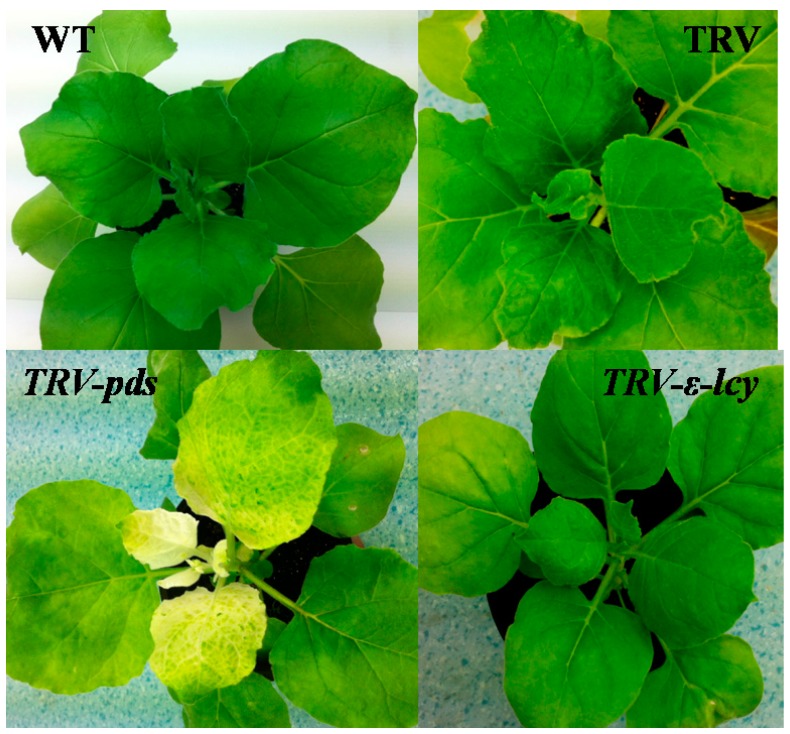
Phenotypes of gene silencing in *N. benthamiana*. WT, plants without infiltration; TRV (tobacco rattle virus), plants infiltrated with the empty vector; *TRV-pds*: plants infiltrated with TRV–PDS; *TRV-ε-lcy*, plants infiltrated with TRV-ε-LCY.

**Figure 6:**
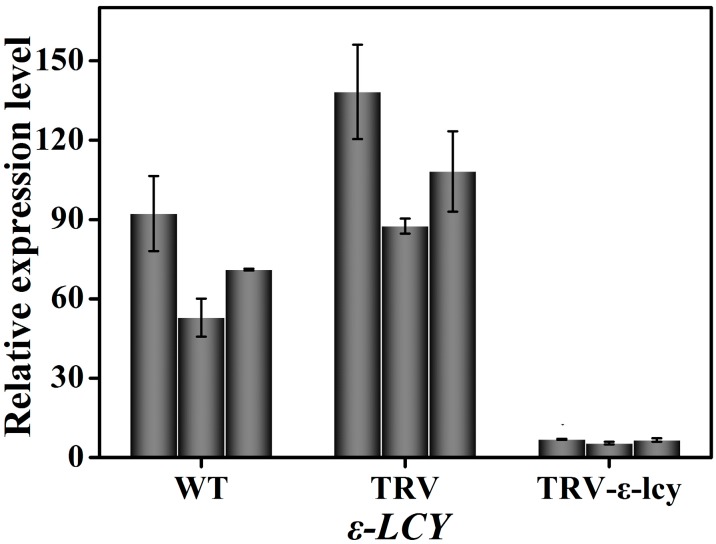
Relative transcript levels of *ε-LCY* in leaves of *TRV-ε-lcy* plants. WT, plants without infiltration; TRV, plants infiltrated with the empty vector; *TRV-ε-lcy*, plants infiltrated with TRV-ε-LCY. The height of each bar represents the mean of three measurements, and the error bars indicate the standard deviation.

**Figure 7:**
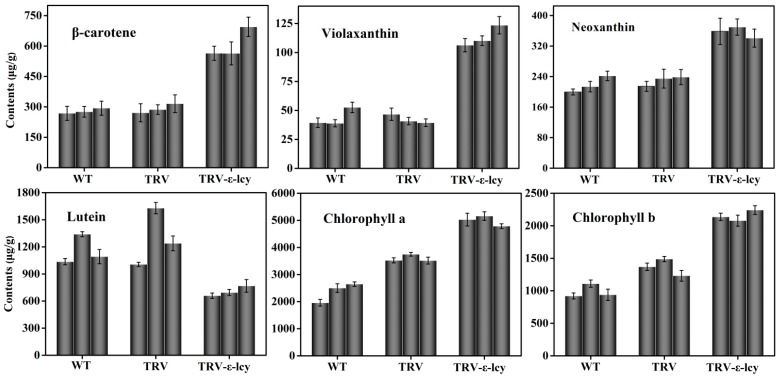
Carotenoids and chlorophyll content in leaves of *TRV-ε-lcy* plants. WT, plants without infiltration; TRV, plants infiltrated with the empty vector; *TRV-ε-lcy*, plants infiltrated with TRV-ε-LCY. The height of each bar represents the mean of three measurements, and the error bars indicate the standard deviation.

TRV-based VIGS, an efficient and convenient technology to silence the expression of endogenous genes in plants, was employed to better understand the physiological function of *ε-LCY* in the carotenoid synthesis process in higher plants. The binary vectors for the expression of TRV–RNA1 (pTRV1) and TRV–RNA2 (pTRV2) were transformed into *Agrobacterium tumefaciens* and infiltrated into leaves of *N. benthamiana* by injection [37,38]. Plants without infiltration (WT) and plants infiltrated with empty vector (TRV) were used as negative controls. *TRV*-*pds*, which silences the *phytoene desaturase* gene (*PDS*) and induces a photo-bleaching phenotype, was used as a positive control to determine the success of gene silencing. In our work, plants injected with TRV–PDS showed a clear photo-bleaching phenotype in top leaves eight days after agroinfiltration, indicating that this TRV–VIGS system was relatively efficient (Figure 5). However, plants infiltrated with TRV-ε-LCY showed no visible phenotype when compared to the negative control plants (Figure 5). Analysis of the *ε-LCY* transcript levels in the *TRV-ε-lcy* plants (Figure 6) showed that transcript levels of *ε-LCY* mRNA in top leaves were significantly reduced compared to control plants. To determine the effects of the reduced *ε-LCY* expression in *TRV-ε-lcy* plants on carotenoid levels, we analyzed the carotenoid composition of *TRV-ε-lcy* and control plants (Figure 7). Levels of β-carotene, violaxanthin, neoxanthin and chlorophyll a and b were found to increase, in *TRV-ε-lcy* plants as compared to WT and TRV control plants. The *TRV-ε-lcy* plants had significantly lower levels of lutein than the control plants.

**Figure 8:**
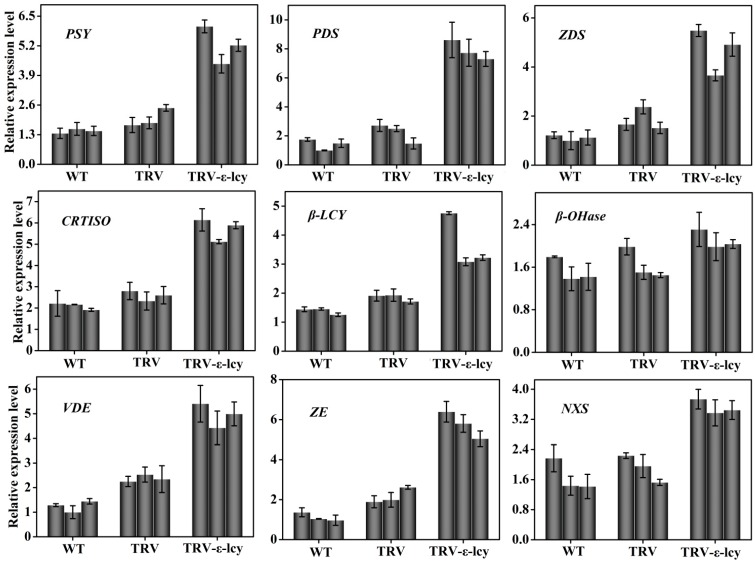
Relative transcript levels of the genes in the carotenoid biosynthetic pathway in the leaves of *TRV-ε-lcy* plants. WT, plants without infiltration; TRV, plants infiltrated with the empty vector; *TRV-ε-lcy*, plants infiltrated with TRV-ε-LCY. The height of each bar represents the mean of three measurements, and the error bars indicate the standard deviation.

Analysis of the transcript levels of the gene for the enzymes in the carotenoid biosynthetic pathway up- and down-stream of the *ε-LCY* branch point indicated that transcript levels of *PSY*, *PDS*, *ZDS*, *CRTISO*, *β-LCY*, *VDE*, *ZE* and *NXS* were all significantly elevated as compared to control plants (Figure 8). The exception was the *β-OHase* gene, which showed a marginal upward trend in expression. These results suggested that the relative activities of ε-cyclase *versus* β-cyclase could determine the flow of carotenoid biosynthetic substrate from lycopene to either α-carotene or β-carotene. Regulation of the two types of lycopene cyclization could therefore be a major mechanism that controls carotenoid composition *in vivo* in higher plants.

### 2.5. Chlorophyll Fluorescence Analysis

**Figure 9:**
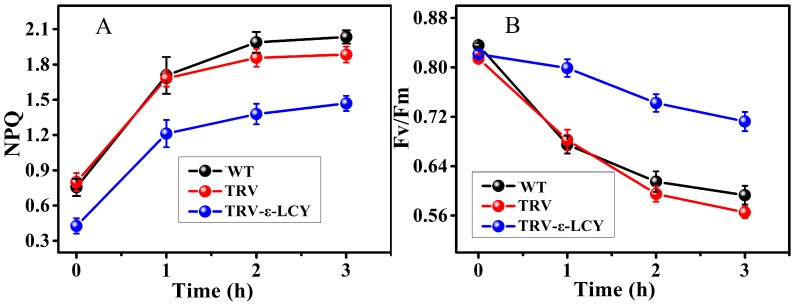
The effects of low temperature and irradiance stress on non-photochemical quenching (NPQ) (**A**) and *F*v/*F*m (**B**) in leaves of *TRV-ε-lcy* plants. WT, plants without infiltration; TRV, plants infiltrated with the empty vector; *TRV-ε-lcy*, plants infiltrated with TRV-ε-LCY. The height of each bar represents the mean of six measurements, and the error bars indicate the standard deviation.

Chlorophyll fluorescence was measured to further analyze the function of *ε-LCY in vivo*. The dissipation of excess energy was monitored by measuring the development of non-photochemical quenching (NPQ). As shown in Figure 9A, NPQ of plants was reduced significantly after infiltration of *TRV-ε-lcy* under normal growth conditions. Under low-temperature and low-irradiation stress, NPQ significantly increased during the first 1 h of treatment and then gradually increased after 1 h in WT, TRV and *TRV-ε-lcy* plants. However, NPQ of the *TRV-ε-lcy* plants remained lower compared to WT and TRV plants, which suggested that less energy was dissipated in a non-photochemical way in the *TRV-ε-lcy* plants.

No difference in (maximal photochemical efficiency of photosystem II) *F*v/*F*m was observed among the WT, TRV and *TRV-ε-lcy* plants under normal growth conditions (Figure 9B). Low-temperature and low-irradiation stress leads to photoinhibition of photosystem II. Under these stresses, *F*v/*F*m decreased in WT, TRV and *TRV-ε-lcy* plants. Unexpectedly, *TRV-ε-lcy* plants had higher *F*v/*F*m compared to WT and TRV plants, indicating that *ε-LCY* silencing can enhance the photosynthetic capacity of *TRV-ε-lcy* plants, which might be an explanation for the lower amount of NPQ and photoinhibition in these plants.

## 3. Discussion

The *ε-LCY* genes of many plant species, such as *Brassica napus* [14], *Zea mays* [15], *Arabidopsis* [18] and *Solanum tuberosum* [26], have been cloned and characterized. This is encoded by a single gene in plants, such as *Arabidopsis* [39] and *Zea mays* [23]. Likewise, the existence of one *ε-LCY* gene in the progenitor diploid *Nicotiana* species, *N. sylvestris* and *N. tomentosiformis*, corresponds to those model plants. In contrast, there are two *ε-LCY* genes in the tetraploid cultivated tobacco plants examined in this study, one of which might have originated from the tobacco progenitor, *N. sylvestris*, and the other from *N. tomentosiformis*. Capillary electrophoresis analysis, PCR fragment and random sequencing results were combined together reveal the copy number for the *ε-LCY* gene in the tobacco genome. According to the results of evolutionary rate analysis, *Ntε-LCY2* has evolved more than twelve times faster than *Ntε-LCY1*. However, the higher mutation rate in the *Ntε-LCY2* coding sequence did not give rise to alteration or transversion of amino acids, which implied that the biological function of the gene did not change as compared to its ancestor. Additionally, the sequence identity between *Ntε-LCY1* and *Ntε-LCY2* was up to 97%, so it was difficult to specifically silence one copy. Therefore, qRT-PCR with conserved primers was applied to analyze the transcriptional expression patterns of *Ntε-LCY in vivo*. There were two interesting characteristics in its transcript expression patterns. First, *Ntε-LCY* transcripts were obviously more abundant in leaves than in other organs, indicating that its function is likely closely related to photosynthesis. In leaves, the amounts of carotenoids, such as β-carotene and lutein, are always proportional to the amounts of chlorophyll, which is crucial for plant photosynthesis. Second, *Ntε-LCY* transcripts displayed the highest expression level in tissues with active vitality, such as samples at the flowering stage. As far as the leaf position is concerned, the 15th leaf (15L) is younger compared to the 10th leaf (10L) and the 5th leaf (5L). Accordingly, *ε-LCY* in 15L displayed the highest expression level in every developmental stage. *Ntε-LCYs* functions mainly in leaves to maintain plant life vitality. Moreover, carotenoids are known to be synthesized in plastids. In this work, we have shown that mature Ntε-LCY protein is located in chloroplasts, which is consistent with its expression patterns in leaves and its biological functions in carotenoid synthesis. However, *Ntε-LCY* transcripts were also detected in stems, roots and flowers, albeit at relatively lower levels. This shows that the physiological function of *ε-LCY* might be required in most plant tissues under natural growth conditions. Plant photosystems are particularly vulnerable to low-temperature and low-irradiation stress, especially photosystem II. It has been reported that there was a five-fold increase in the ratio of *β-LCY* mRNA to *ε-LCY* mRNA in *Arabidopsis* and tomato leaves when the plants were shifted from low light to strong light [40]. The study illustrated that strong light was beneficial for the expression of *β-LCY* gene, while low light availed *ε-LCY* gene expression. Therefore, the *Ntε-LCY* gene was evaluated, and its expression was found to be induced under the low-temperature and low-irradiation stress. The results made us to predict that manipulation of *Ntε-LCY* could change the carotenoids composition and regulate photosynthetic capacity.

The cyclization of lycopene is a key regulatory branching point in the carotenoid biosynthetic pathway. The enzyme activities of β-LCY and ε-LCY affect carotenoid composition and plant growth and development [16,24,41]. Pogson *et al.* [24] found that the genetic lesions in *β-LCY* were lethal in *Arabidopsis*, indicating the requirement for bicyclic carotenoids for plant viability. Transgenic *Arabidopsis* expressing *β-LCY* from *Salicornia europaea* exhibited enhanced tolerance to oxidative stress and salt stress [42]. On the contrary, researchers identified that knocking out or knocking down expression of the *ε-LCY* gene did not affect plant viability [14,24,25,26]. In maize, natural variation of *ε-LCY* genes could explain 58% of the variation in the content of lutein and β-carotene among different cultivars [23]. In sweet potato calli, downregulation of *ε-LCY* transcript levels increased carotenoid content via the β-branch-specific pathway, and this increase enhanced salt-stress tolerance [25]. Similarly, in potato tubers, silencing of the *ε-LCY* gene raised β-carotene content and caused a minor decrease in lutein [26]. In *Brassica napus*, suppression of *ε-LCY* gene in seeds resulted in higher β-carotenoid content, but unexpectedly increased lutein [14]. There are always some differences in the function of *ε-LCY* genes in different tissues in higher plants. In this work, VIGS, a rapid and reliable gene silencing technology, was employed to characterize the function of the *Ntε-LCY* gene in the leaves of *N. benthamiana*. A conserved fragment of two *Ntε-LCY* cDNAs was selected as an RNAi effective element, and the silencing efficiency achieved up to 90%–95% reduction in transcript levels. As expected, β-branch carotenoid concentrations all increased in the leaves of *TRV-ε-lcy* plants and the lutein content decreased. Proportionally, chlorophyll content increased in the leaves, indicating that the ratio of phytosynthetic pigments in plants was under strict genetic and physiological regulation. Results of VIGS experiments showed that *ε-LCY* was rate-limiting for lutein synthesis and could regulate the ratio of carotenoids in tobacco leaves. Furthermore, gene transcriptional analysis involved in the carotenoid biosynthesis pathway indicated that the increased expression of *PSY*, *PDS*, *ZDS*, *CRTISO*, *β-LCY*, *ZE*, *VDE* and *NXS* genes up- and down-stream of *ε-LCY* were determined in *TRV-ε-lcy* lines. These results provided an explanation for the variation in carotenoid content and suggested that there may be a feedback mechanism in the carotenoid pathway.

Carotenoids and chlorophylls are essential components of plant photosystems and are especially critical for the light harvesting complex. As mentioned previously, overexpression of *ε-LCY* in leaves of *Arabidopsis* resulted in an increase of lutein and the reduction of β-carotene and xanthophylls, but caused an increase in NPQ. It has been reported that α- and β-branch xanthophyll have distinct and complementary roles in the photoprotection mechanisms [43]. Modulation of the xanthophyll composition can greatly affect photosystem assembly, light harvesting, photoprotection and the ability of plants to respond to stress [44]. Kalituho *et al.* [45] found that replacement of lutein by violaxanthin in the *lut2npq1* mutant led to a pronounced reduction of growth under high light conditions, which indicated an important photoprotective role for lutein. Meanwhile, the high susceptibility of *lut2npq2* to photoinhibition, in comparison with *npq2*, further indicated that the photoprotective function of zeaxanthin is abolished in the absence of lutein. These reports all demonstrated that lutein plays an irreplaceable role in photoprotection in plants. When *ε-LCY* is silenced in tobacco plants, a balance of carotenoids is formed, so we wonder if there was a new balance of photosynthesis *in vivo*. The photosynthetic properties of *TRV-ε-lcy* plants were studied by measuring chlorophyll fluorescence, and the results showed that NPQ increased in both *TRV-ε-lcy* and WT plants under low-temperature and low-irradiation stress. However, the NPQ of *TRV-ε-lcy* plants was always lower than that of WT plants. Three possibilities may explain this observation. First, when *ε-LCY* was silenced in tobacco plants, the contents of carotenoids and chlorophylls increased, so there should be an improvement in photosynthetic ability in leaves, as *F*v/*F*m increased. Increasing of photosynthetic capacity would mean that more energy could be consumed by photosynthetic electron transport chain. That is to say, there was less excess energy that needed to be dissipated by non-photochemical quenching. Therefore, there might be more β-xanthophylls *in vivo*, but these pigments did not need to function effectively. Second, less lutein might affect the photosystem assembly and photoprotection mechanism, as α-xanthophylls are also important for photosystem protection. Third, the downstream product of the β-branch carotenoids is ABA, an essential plant hormone that is crucial for plant development and stress resistance. Carotenoids of the β-branch could generate more ABA, which might regulate the stress tolerance of plants. In the stress treatment experiment, *ε-LCY* transcript expression increased in response to the stress. However, our results from the VIGS experiments showed that lower *ε-LCY* expression was beneficial for photosynthesis. Probably when stress was transient, plant needed to synthesize more lutein for NPQ to dissipate extra energy. When the duration of the stress was prolonged, *ε-LCY* transcript expression levels decreased gradually after 24 h of stress treatment to help to generate more ABA.

Through reducing the transcript level of *ε-LCY*, more carotenoids could be synthesized in *N. benthamiana*, which might provide an efficient strategy to manipulate tobacco carotenoids. In combination with other genes, such as *PSY*, a genetic system for the metabolic engineering of the carotenoid synthetic pathway in *N. tabacum* could be generated. Some other metabolites, such as fatty acids and polysaccharides, should be checked to reflect the gene additive effect. Furthermore, genetic mechanism under the regulatory role of *ε-LCY* on carotenoid content would be investigated to realize the genetic regulatory networks controlling plant carotenoid content.

## 4. Experimental Section

### 4.1. Plant Materials

Seeds of *N. tabacum*, *N. sylvestris*, *N. tomentosiformis* and *N. benthamiana* were retained in the laboratory. *N. benthamiana* was cultured in a greenhouse at 23 °C with a photo-cycle of 16 h light/8 h dark at the National Tobacco Gene Research Center of the Zhengzhou Tobacco Research Institute, Zhengzhou, China. *N. sylvestris* and *N. tomentosiformis* were grown in a greenhouse at 28 °C with the other conditions the same as those of *N. benthamiana*. *N. tabacum* used for clone and expression analysis samples was cultivated in the experimental farm in Yunnan province. The leaves, stems, roots and flowers of tobacco were collected and stored at −80 °C. For low temperature stress and irradiance treatments, the plants were exposed to 4 °C with 100 μmol·photons·m^−2^·s^−1^ irradiance.

### 4.2. BY-2 Cell Culture Conditions and Growth Measurement

BY-2 tobacco cells (*Nicotiana tabacum* L. cv. Bright Yellow 2) were grown in modified Linsmaier and Skoog medium containing 4.3 g/L MS salts supplemented with 0.2 mg/L 2,4-Dichlorophenoxyacetic acid (2,4-D), 0.18 g/L KH_2_PO_4_, 0.1 g/L myo-inositol, 1 mg/L thiamine HCl and 30 g/L sucrose. The medium was adjusted to pH 5.8 with 1 mol/L KOH before autoclaving. The cells were propagated at 22 °C under continuous shaking (100 rpm) for 7 days.

### 4.3. Genomic DNA and RNA Isolation and cDNA Synthesis

Total RNA and DNA were isolated using RNeasy Plant Mini Kits and DNeasy Plant Mini Kits (Qiagen, Hilden, Germany), respectively. To generate templates for gene cloning and qRT-PCR analysis, first-strand cDNA synthesis was performed using total RNA treated with DNase using the SuperScript First-Strand Synthesis System (Takara, Otsu, Japan) primed with oligo(dT)_18_ according to the manufacturer’s instructions.

### 4.4. Cloning of ε-LCY and Vector Construction

The *ε-LCY* gene was amplified by PCR from cDNA and genomic DNA of *N. tabacum*, *N. sylvestris* and *N. tomentosiformis* leaves with the primers ε-LCY-F (5'-ATGGATTGTATTGGAGCTCGAAAT-3') and ε-LCY-R (5'-CTAAAATGTAAGATAAGTTCTTATCA-3'). The amplified products were cloned into the pMD19-T vector (Takara, Otsu, Japan) and then sequenced. To construct the subcellular localization vector, the specific primers were designed from the sequence of *Ntε-LCY*, which contained the following SalI and BamHI restriction sites: 5'-TAGTCGACATGGATTGTATTGGAGCTCGAAAT-3' and 5'-GCGGATCCAAATGTAAGATAAGTTCTTATCA-3'. The PCR product was digested with SalI and BamHI and ligated into the pJIT163-hGFP vector (From Professor Daowen Wang’s Lab at the Institute of Genetics and Developmental Biology in Beijing, China).

To investigate the copy number of *ε-LCY* genes in *N. tabacum*, we made use of intron length polymorphisms and designed a pair of intron flanking (IF) primers: 5'-GAAGACGAGTTCAAAGGTCGTAATC-3' and 5'-ATGCTTGAAGCCCAAGATCTG-3'.

For the functional analysis, a fragment of 602 bp was selected and amplified from 334 to 935 bp of *Nt*ε-LCY, and the specific primers of the VIGS vector were designed with KpnI and XhoI restriction sites: 5'-GCGGTACCGTCCTGCTGGTCTTGCTCTTGCT-3' and 5'-GCCTCGAGGACTCCTGTTTTAGTCATGGGCATG-3'. The fragment was subsequently cloned into the pTRV2 vector supplied by Professor Dawei Li (Chinese Agricultural University, Beijing, China).

### 4.5. Evolutionary Analysis of ε-LCY

The *ε-LCY* protein sequences in *N. tabacum* were aligned with those in *N. sylvestris* and *N. tomentosiformis* using Clustal W [46]. Coding sequence alignments were carried out under the guidance of protein alignments through an in-house Perl script. The *K*a (the number of substitutions per nonsynonymous site) and *K*s (the number of substitutions per synonymous site) values were calculated by using *K*a*K*s_calculator [47] with the method of Nei and Gojobori. The evolutionary rate was estimated as previously described [48].

### 4.6. BY-2 Suspension Cell Bombardment and Fluorescence Microscopy

Transient transformation of BY-2 suspension cells was performed with the Bio-Rad PDS1000/He system (Bio-Rad, Hercules, CA, USA) by following the manufacturer’s protocol. Gold particles (diameter: 1–2 μm) with a microcarrier loading quantity of 0.5 mg gold/DNA were chosen for the particle bombardment. Seven-day-old BY-2 cells were bombarded at a pressure of 1100 p.s.i./shot. After bombardment, BY-2 cells were cultivated for 12 h in the dark at room temperature prior to subcellular localization characterization. The bombarded BY-2 cells were visualized with confocal microscopy (Leica, Wetzlar, Germany) with excitations at 475–490 and 545–565 nm and emission at 510–530 and 585–620 nm.

### 4.7. Agrobacterium Transformation and Infiltration

The pTRV1, pTRV2, pTRV2-PDS and pTRV2-ε-LCY vectors were transformed into *A. tumefaciens* strain GV3101 by heat shock methods. *Agrobacterium* cultures containing pTRV1, pTRV2, pTRV2-PDS and pTRV2-ε-LCY were grown overnight at 28 °C in LB medium containing antibiotics (50 μg/mL kanamycin, 25 μg/mL rifampicin), 10 mM 2-(*N*-morpholine) ethane sulfonic acid (MES) and 20 μM acetosyringone. The cultures were centrifuged at 5000 rpm at room temperature for 10 min and resuspended in infiltration medium (containing 10 mM MES, 10 mM MgCl_2_ and 200 μM acetosyringone in LB medium) to a final OD_600_ of 1.0. Cell suspensions were incubated at room temperature for at least 3 h without shaking before injection. A mixture of *Agrobacterium* cultures containing pTRV1 and pTRV2 (negative control, named as TRV) or its derivative (pTRV2-PDS, positive controls, named as TRV-PDS, or pTRV2-ε-LCY, named as *TRV-ε-lcy*) constructs in a 1:1 ratio were injected into the leaves of four-week-old *N. benthamiana* plants using a needleless syringe.

### 4.8. Gene Expression Analysis

qRT-PCR was employed to analyze the relative expression levels of the various genes. Briefly, the reaction was performed with a Fluorescent Quantitative PCR Detector (Bio-Rad, Hercules, CA, USA). SYBR Green real-time PCR Master Mix (Qiagen, Hilden, Germany) was used. The 26s RNA gene was used as a reference gene to eliminate the error of reverse transcription reactions. qRT-PCR products were assessed by melting curve and gel electrophoresis to ensure the specificity of the amplification in the reactions. Three technical replicates were carried out for each biological sample. Conditions for qRT-PCR cycling were 95 °C for 3 min, 95 °C for 20 s, 60 °C for 20 s, 40 cycles. The relative expression level of each gene was calculated using the 2^−ΔΔ*C*q^ method. Primers used in the qRT-PCR analysis of the expression levels of *ε-LCY* and other carotenoid biosynthesis related genes are listed in Table 1.

**Table 1:** Gene-specific primers used for qRT-PCR analysis.

Gene	Primer	Primer Sequence
*PSY*	PSY-Q-F	TGTTGGAGAAGATGCCAGAAGAG
	PSY-Q-R	ATAAGCAATAGGTAAGGAAATTAGCTTC
*PDS*	PDS-Q-F	ATAAACCCTGACGAGCTTTC
	PDS-Q-R	AATATGTTCAACAATCGGCAT
*ZDS*	ZDS-Q-R	TGAAATAGGGGAGCTTGATTTCCGC
	ZDS-Q-F	GAGCATATGCGACAGGATCCCAC
*CRTISO*	CRTISO-Q-F	CGTGTACACCGAGAATATGATG
	CRTISO-Q-R	GTAGGCGAGAGTCAAGCACTC
*β-LCY*	β-LCY-Q-F	GATGACAATACAACTAAAGATCTTGATAG
	β-LCY-Q-R	CATAAGCTACTTGATATCCAGGAT
*ε-LCY*	ε-LCY-Q-F	CAGGAGTCTTTTTCGAGGAAACTTG
	ε-LCY-Q-R	GTGTTCCAAGCTTGAGTTGAGAT
*β-OHase*	β-OHase-Q-F	ATGGCCGCCAGCAGAATTTC
	β-OHase-Q-R	CTCAATTTTCATTTCAATCTCCTCTGTC
*VDE*	VDE-Q-F	ATGATGCATGGGATGGATATG
	VDE-Q-R	CGTTGGAGCTCTTTAAAACCTTC
*ZE*	ZE-Q-F	GTGGTGGGATTGGAGGGTTAGTG
	ZE-Q-R	AGGATCTGCTGCAAAGTCATGC
*NXS*	NXS-Q-F	GCCGGGCTCTATTCGACGTGAT
	NXS-Q-R	ACTGACTCTACCATATGGTCTTCCCAAAT
*26S-RNA*	26S-RNA-Q-F	GAAGAAGGTCCCAAGGGTTC
	26S-RNA-Q-R	TCTCCCTTTAACACCAACGG

### 4.9. Carotenoid Extraction and Quantification

Carotenoids were extracted from 200 mg of freeze dried leaf samples with 25 mL of acetone. The samples were sonicated for 20 min, filtered through 0.22 μm cellulose acetate filters (Shanghai Chuding Analytical Instruments Ltd., Shanghai, China), and analyzed by HPLC.

For HPLC analysis, the carotenoids were separated on an Agilent 1100 HPLC system with a C_18_ column (3.9 × 150 mm, 3 μm; Waters Corporation, Milford, MA, USA) and detected with a diode array detector (DAD) at 448 and 428 nm. Solvent A consisted of isopropanol. Solvent B consisted of 80% acetonitrile–water.

### 4.10. In Vivo Fluorescence and Non-Photochemical Quenching (NPQ) Measurements

Non-photochemical quenching of chlorophyll fluorescence and PSII yield (*F*v/*F*m) were measured for leaves at room temperature with a Dual-PAM-100 fluorimeter (Walz, Effeltrich, Germany). NPQ, was calculated according to the equation, NPQ = (*F*m − *F*'m)/*F*'m. *F*m is the maximum Chl fluorescence from dark-adapted leaves; *F*'m is the maximum Chl fluorescence under actinic light exposition.

### 4.11. Statistical Analysis

All data were expressed as the mean ± SD of three independent replicates. The statistical analysis was performed with SPSS for Windows Version 16.0 (SPSS Inc., Chicago, IL, USA), and statistical analyses were made using one-way ANOVA tests. Values of *p* ≤ 0.05 were considered to be statistically significant.

## 5. Conclusions

In conclusion, this work has generated new information about *Ntε-LCY* genes and their evolution in *N. tabacum*, and their functions were examined in *N. benthamiana* using TRV-VIGS technology. Suppression of *ε-LCY* expression was found to alleviate photoinhibition of PSII in VIGS plants under low-temperature and low-irradiation stress. Our results provide insight into the regulatory role of *ε-LCY* in plant carotenoid biosynthesis and suggest a role for *ε-LCY* in the modulation of low temperature and low-irradiation stress responses. Our work lays the groundwork for the future manipulation of carotenoid composition in plants.

## Supplementary Files

Supplementary File 1

Supplementary File 2

Supplementary File 3

Supplementary File 4
