# Supplementary material for: Molecular Cloning and Functional Characterization of the Lycopene ε-Cyclase Gene via Virus-Induced Gene Silencing and Its Expression Pattern in Nicotiana tabacum
Source: Int J Mol Sci. 2014 Aug 22;15(8):14766–85. doi: 10.3390/ijms150814766 (PMC4159881; doi:10.3390/ijms150814766)
Supplement: Supplementary File 1 [file ijms-15-14766-s001.pdf]

Supplementary File

Multipile alignment of ε-LCY proteins from higher plants. Amino acid sequence identities. Comparation of amino sequences of *Nicotiana tabacum* (1: Ntε-LCY1), *N. sylvestris* (2: Nsyε-LCY), *N. tomentosiformis* (3: Ntomε-LCY), *Solanum tuberosum* (4: Stε-LCY), *Solanum lycopersicum* (5: Slε-LCY), *Arabidopsis* (6: Atε-LCY).

```
1 : MDCIGARNFSTMAVFTCPRFKSLGRKRIMPRKKQPFWPIH-MKVKCS-----GSDSCVVVKEDFADEEDYIKAGGSELVFVQMQQNKDMDLQSKLSDKLRQISSAG : 100
2 : MECIGARNFATMAVFTCPRFKSLGRKRIMPRKKQPFWPIH-MKVKCS-----GSDSCVVVKEDFADEEDYIKAGGSELVFVQMQQNKDMDLQSKLSDKLRQISSAG : 100
3 : MECIGARNFATMAVFTCPRFKSLGRRRIMPRKKQPFWPIH-MQVKCS-----GNESCVVVKEDFADEEDYIKAGGSELVFVQMQQNKDMDLQSKLSDKLRQISSTG : 100
4 : MECVGAQNVGAMAVFTRPRLKPLVGRRIMPRKKQSFWPMSSMQVKCNS--SSGSESCVVDKEDFADEEDYIKAGGSQLIFVQMQQKKDMDQQSKLSDELRQIS-AG : 103
5 : MECVGAQNVGAMAVFTRPRLKPLVGRRVMPRKKQSFWRMSSMKVKCNS--SSGSDSCVVDKEDFADEEDYIKAGGSQLVFVQMQQKKDMDQQSKLSDELRQIS-AG : 103
6 : MECVGARNFAAMAVSTFPSWSCRRKFPVVKRYSYRNIRFGLCSVRASGGGSSGSESCVAVREDFADEEDFVKAGGSEILFVQMQQNKDMDEQSKLVDKLPPIS-IG : 105

1 : QTILDLVVIGCGPAGLALAAESAKLGLNVGLVGPDLPFTNNYGVWEDEFKDLGLQACIEHVWSDTIVYLDDADPILIGRAYGRVSRHLLHEELLKRCVEAGVLYLN : 206
2 : QTILDLVVIGCGPAGLALAAESAKLGLNVGLVGPDLPFTNNYGVWEDEFKDLGLQACIEHVWSDTIVYLDDADPILIGRAYGRVSRHLLHEELLKRCVEAGVLYLN : 206
3 : QTILDLVVIGCGPAGLALAAESAKLGLNVGLVGPDLPFTNNYGVWEDEFKDLGLQACIEHVWRDTIVYLDDADPILIGRAYGRVSRHLLHEELLKRCVEAGVLYLN : 206
4 : QTVLDLVVIGCGPAGLALAAESAKLGLNVGLVGPDLPFTNNYGVWEDEFKDLGLQACIEHVWRDTIVYLDDDDPILIGRAYGRVSRHLLHEELLKRCVEAGVLYLN : 209
5 : QTVLDLVVIGCGPAGLALAAESAKLGLNVGLVGPDLPFTNNYGVWEDEFKDLGLQACIEHVWRDTIVYLDDDEPILIGRAYGRVSRHFLHEELLKRCVEAGVLYLN : 209
6 : DGALDLVVIGCGPAGLALAAESAKLGLKVGLIGPDLPFTNNYGVWEDEFNDLGLQKCIEHVWRETIVYLDDDKPITIGRAYGRVSRRLLHEELLRRCVESGVSYLS : 211

1 : SKVDRIVESTSGHSLVECEGDIVIPCRFVTVASGAASGKFLQYELGGPRVSVQTAYGVEVEVDNNPYDPSLMVFMDYRDYVRHDAQSLEAKYPTFLYAMPMTKTGV : 312
2 : SKVDRIVESTSGHSLVECEGDIVIPCRFVTVASGAASGKFLQYELGGPRVSVQTAYGVEVEVDNNPYDPSLMVFMDYRDYVRHDAQSLEAKYPTFLYAMPMTKTRV : 312
3 : SKVDRIVESTSGHSLVECEGDIVIPCRFVTVASGAASGKFLQYELGGPRVSVQTAYGVEVEVDNNPYDPSLMVFMDYRDYVRHDAQSLEAKYPTFLYAMPMTKTRV : 312
4 : SKVDRIVEATNGHSLVECEGDVVIPCRFVTVASGAASGKFLQYELGGPRVSVQTAYGVEVEVDNNPFDPSLMVFMDYRDYVRHDAQSLEAKYPTFLYAMPMSPTRV : 315
5 : SKVDRIVEATNGQSLVECEGDVVIPCRFVTVASGAASGKFLQYELGGPRVSVQTAYGVEVEVDNNPFDPSLMVFMDYRDYVRHDAQSLEAKYPTFLYAMPMSPTRV : 315
6 : SKVDSITEASDGLRLVACDDNNVIPCRLATVASGAASGKLLQYEVGGPRVCVQTAYGVEVEVENSPYDPDQMVFMDYRDYTNEKVRSLEAEYPTFLYAMPMTKSRL : 317

1 : FFEETCLASKDAMPFDLLKKKLMLRLNTLGIKIKKIYEEEWSYIPVGGSLPNTEQKTLAFGAAASMVHPATGYSVVRSLSEAPKCASVLANILRQNHVKNMITSSS : 418
2 : FFEETCLASKDAMPFDLLKKKLMLRLNTLGIKIKKIYEEEWSYIPVGGSLPNTEQKTLAFGAAASMVHPATGYSVVRSLSEAPKCASVLANILRQNHVKNMITSSS : 418
3 : FFEETCLASKDAMPFDLLKKKLMLRLNTLGVRIKQIYEEEWSYIPVGGSLPNTEQKTLAFGAAASMVHPATGYSVVRSLSEAPKCASVLANILRQNHVKNMLTSSS : 418
4 : FFEETCLASKDAMPFDLLKKKLMLRLNTLGVRIKEIYEEEWSYIPVGGSLPNTEQKTLAFGAAASMVHPATGYSVVRSLSEAPKCASVLANILRQNHSKNMLTSSS : 421
5 : FFEETCLASKDAMPFDLLKKKLMLRLNTLGVRIKEIYEEEWSYIPVGGSLPNTEQKTLAFGAAASMVHPATGYSVVRSLSEAPKCASVLANILRQHYSKNMLTSSS : 421
6 : FFEETCLASKDVMPFDLLKTKLMLRLDTLGIRILKTYEEEWSYIPVGGSLPNTEQKNLAFGAAASMVHPATGYSVVRSLSEAPKYASVIAEILREETTKQINSN-- : 421

1 : ATSISTQAWNTLWPQERKRQRSFFLFGLALILQLDIEGIRSFFRAFFRVPKWMWQGFLGSSLSSADLMLFAFYMFIIAPNDMRKGLIRHLLSDPTGATMIRTYLTF : 524
2 : ATSISTQAWNTLWPQERKRQRSFFLFGLALILQLDIEGIRSFFRAFFRVPKWMWQGFLGSSLSSADLMLFAFYMFIIAPNDMRKGLIRHLLSDPTGATMIRTYLTF : 524
3 : TTSISTQAWNTLWPQERKRQRSFFLFGLALILQLDIEGIRSFFRAFFLVPKWMWQGFLGSSLSSADLMLFAFYMFIIAPNDMRKGLIRHLLSDPTGATMIRTYLTF : 524
4 : TPSISTQAWNTLWPQERKRQRSFFLFGLALILQLDIEGIRSFFRAFFRVPKWMWQGFLGSSLSSADLMLFAFYMFIIAPNDMRRGLIRHLLSDPTGATLIRTYLTF : 527
5 : IPSISTQAWNTLWPQERKRQRSFFLFGLALILQLDIEGIRSFFRAFFRVPKWMWQGFLGSSLSSADLMLFAFYMFIIAPNDMRKGLIRHLLSDPTGATLIRTYLTF : 527
6 : ---ISRQAWDTLWPPERKRQRAFFLFGLALIVQFDTEGIRSFFRTFFRLPKWMWQGFLGSTLTSGDLVLFALYMFVISPNNLRKGLINHLISDPTGATMIKTYLKV : 524
```

Identities of Ntε-LCY1 with other ε-LCYs in higher plants.

**Amino Acid**	**Nsyε-LCY**	**Ntomε-LCY**	**Stε-LCY**	**Slε-LCY**	**Atε-LCY**
Identity (%)	99.4	97.1	90.9	90.3	72.6
