# Supplementary material for: Molecular Cloning and Functional Characterization of the Lycopene ε-Cyclase Gene via Virus-Induced Gene Silencing and Its Expression Pattern in Nicotiana tabacum
Source: Int J Mol Sci. 2014 Aug 22;15(8):14766–85. doi: 10.3390/ijms150814766 (PMC4159881; doi:10.3390/ijms150814766)

Supplementary File

Electropherograms of fragment amplified in genomic DNA of *N. sylvestris* (**A**) and *N. tomentosiformis* (**B**) using intron flanking (IF) primers labeled by FAM.

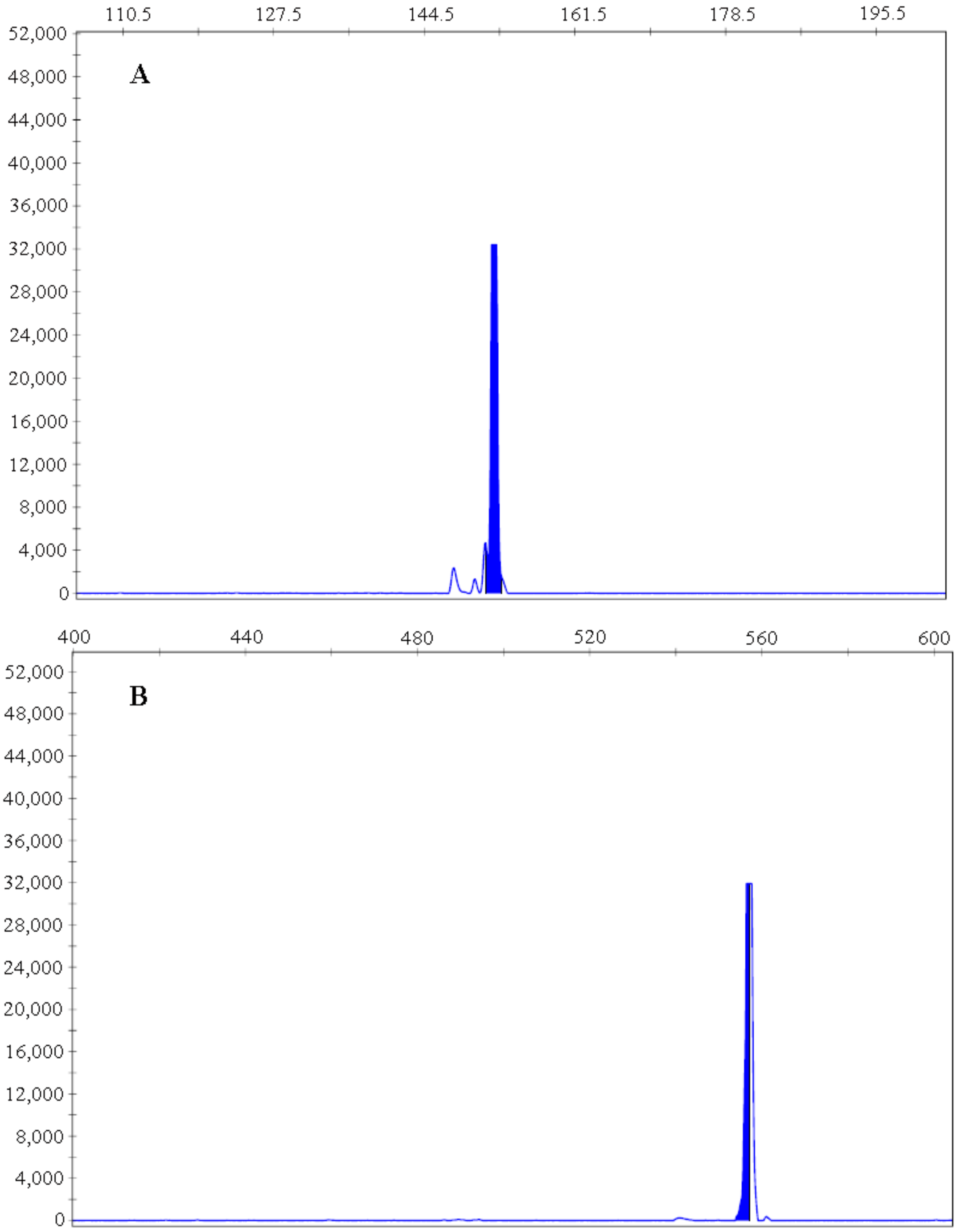

Supplement: Supplementary File 3 [file ijms-15-14766-s003.pdf]
