# Supplementary material for: Molecular Cloning and Functional Characterization of the Lycopene ε-Cyclase Gene via Virus-Induced Gene Silencing and Its Expression Pattern in Nicotiana tabacum
Source: Int J Mol Sci. 2014 Aug 22;15(8):14766–85. doi: 10.3390/ijms150814766 (PMC4159881; doi:10.3390/ijms150814766)
Supplement: Supplementary File 4 [file ijms-15-14766-s004.pdf]

Supplementary File

Coding sequence comparison between *Ntε-LCY1* (Nt-1) and *Nsyε-LCY* (Nsyl)

```
Nsyl : ATGGATTGTATTGGAGCTCGAAATTTTGCTACAATGGCGGTTTTTACGTGTCCGAGATTCAAATCATTAGGAAGAAAGAGAATTATGCCA :   90
Nt-1 : ATGGATTGTATTGGAGCTCGAAATTTTTCTACAATGGCGGTTTTTACGTGTCCGAGATTCAAATCATTAGGAAGAAAGAGAATTATGCCA :   90

Nsyl : AGAAAAAAGCAACCATTTTGGCCTATACATATGAAAGTGAAGTGTAGTGGAAGTGATAGTTGTGTAGTGGTTAAAGAAGATTTTGCTGAT :  180
Nt-1 : AGAAAAAAGCAACCATTTTGGCCTATACATATGAAAGTGAAGTGTAGTGGAAGTGATAGTTGTGTAGTGGTTAAAGAAGATTTTGCTGAT :  180

Nsyl : GAAGAAGATTACATAAAAGCTGGTGGTTCAGAACTTGTTTTTGTTCAAATGCAGCAGAATAAAGACATGGATCTACAGTCTAAGCTTTCT :  270
Nt-1 : GAAGAAGATTATATAAAAGCTGGTGGTTCAGAACTTGTTTTTGTTCAAATGCAGCAGAATAAAGACATGGATCTACAGTCTAAGCTTTCT :  270

Nsyl : GATAAGTTGCGACAAATATCATCAGCTGGACAAACTATACTGGATTTAGTGGTCATTGGCTGTGGTCCTGCTGGTCTTGCTCTTGCTGCG :  360
Nt-1 : GATAAGTTGCGACAAATATCATCAGCTGGACAAACTATACTGGATTTAGTGGTCATTGGCTGTGGTCCTGCTGGTCTTGCTCTTGCTGCG :  360

Nsyl : GAGTCTGCTAAACTCGGGTTGAACGTGGGGCTCGTTGGTCCTGATCTTCCTTTCACAAATAACTATGGTGTCTGGGAAGACGAGTTCAAA :  450
Nt-1 : GAGTCTGCTAAACTCGGGTTGAACGTGGGGCTCGTTGGTCCTGATCTTCCTTTCACAAATAACTATGGTGTCTGGGAAGACGAGTTCAAA :  450

Nsyl : GATCTTGGGCTTCAAGCATGCATTGAACATGTTTGGAGTGATACCATTGTATATCTTGATGATGCCGATCCAATTCTTATTGGACGTGCT :  540
Nt-1 : GATCTTGGGCTTCAAGCATGCATTGAACATGTTTGGAGTGATACCATTGTATATCTTGATGATGCCGATCCAATTCTTATTGGACGTGCT :  540

Nsyl : TATGGAAGAGTTAGTCGCCATTTACTGCATGAGGAGTTACTCAAAAGGTGTGTGGAGGCAGGTGTTTTATATCTTAACTCGAAAGTGGAT :  630
Nt-1 : TATGGAAGAGTTAGTCGCCATTTACTGCATGAGGAGTTACTCAAAAGGTGTGTGGAGGCAGGTGTTTTATATCTTAACTCGAAAGTGGAT :  630

Nsyl : AGGATCGTTGAGTCCACAAGTGGCCACAGTCTTGTAGAGTGCGAGGGCGACATTGTCATTCCTTGCAGGTTTGTCACTGTTGCATCTGGA :  720
Nt-1 : AGGATCGTTGAGTCCACAAGTGGCCACAGTCTTGTAGAGTGCGAGGGCGACATTGTCATTCCTTGCAGGTTTGTCACTGTTGCATCTGGA :  720

Nsyl : GCCGCCTCAGGGAAATTCTTGCAGTATGAGTTGGGAGGTCCTCGGGTTTCTGTTCAAACAGCTTATGGAGTGGAAGTTGAGGTCGATAAC :  810
Nt-1 : GCCGCCTCAGGGAAATTCTTGCAGTATGAGTTGGGAGGTCCTCGGGTTTCTGTTCAAACAGCTTATGGAGTGGAAGTTGAGGTCGATAAC :  810

Nsyl : AATCCGTATGATCCAAGCCTGATGGTTTTCATGGATTATAGAGACTATGTCAGACACGACGCTCAATCTTTAGAAGCTAAATATCCAACA :  900
Nt-1 : AATCCGTATGATCCAAGCCTGATGGTTTTCATGGATTATAGAGACTATGTCAGACACGACGCTCAATCTTTAGAAGCTAAATATCCAACA :  900

Nsyl : TTTCTTTATGCCATGCCCATGACTAAAACAAGAGTCTTTTTCGAGGAAACTTGTTTGGCTTCAAAAGATGCAATGCCATTTGATCTATTA :  990
Nt-1 : TTTCTTTATGCCATGCCCATGACTAAAACAGGAGTCTTTTTCGAGGAAACTTGTTTGGCTTCAAAAGATGCAATGCCATTTGATCTATTA :  990
```

```
Nsyl : AAGAAAAAACTGATGTTACGATTGAACACATTGGGCATAAAAATTAAAAAAATCTACGAGGAGGAATGGTCTTACATACCAGTTGGTGGA : 1080
Nt-1 : AAGAAAAAACTGATGTTACGATTGAACACATTGGGCATAAAAATTAAAAAAATCTACGAGGAGGAATGGTCTTACATACCAGTTGGTGGA : 1080

Nsyl : TCGTTGCCAAATACAGAGCAGAAAACACTTGCGTTTGGCGCTGCTGCTAGCATGGTTCATCCAGCTACAGGTTATTCAGTTGTCAGATCA : 1170
Nt-1 : TCGTTGCCAAATACAGAGCAGAAAACACTTGCGTTTGGCGCTGCTGCTAGCATGGTTCATCCAGCTACAGGTTATTCAGTTGTCAGATCA : 1170

Nsyl : CTGTCCGAGGCGCCAAAATGCGCCTCCGTACTTGCAAATATTTTAAGACAAAATCATGTCAAGAACATGATTACCAGTTCAAGTGCCACA : 1260
Nt-1 : CTGTCCGAGGCGCCAAAATGCGCCTCCGTACTTGCAAATATTTTAAGACAAAATCATGTCAAGAACATGATTACCAGTTCAAGTGCCACA : 1260

Nsyl : AGTATCTCAACTCAAGCTTGGAACACCCTTTGGCCACAAGAACGAAAAAGGCAACGATCGTTTTTCCTATTTGGATTGGCACTCATATTG : 1350
Nt-1 : AGTATCTCAACTCAAGCTTGGAACACCCTTTGGCCACAAGAACGAAAAAGGCAACGATCGTTTTTCCTATTTGGATTGGCACTCATATTG : 1350

Nsyl : CAGCTGGATATTGAGGGGATTAGGTCATTTTTCCGCGCATTCTTCCGTGTACCAAAATGGATGTGGCAAGGTTTTCTTGGCTCTAGTCTT : 1440
Nt-1 : CAGCTGGATATTGAGGGGATTAGGTCATTTTTCCGCGCATTCTTCCGTGTACCAAAATGGATGTGGCAAGGTTTTCTTGGCTCTAGTCTT : 1440

Nsyl : TCATCAGCAGACCTCATGTTATTTGCCTTCTACATGTTTATTATTGCACCAAATGACATGAGAAAAGGCCTAATCAGACATCTGTTATCT : 1530
Nt-1 : TCATCAGCAGACCTCATGTTATTTGCCTTCTACATGTTTATTATTGCACCAAATGACATGAGAAAAGGCCTAATCAGACATCTGTTATCT : 1530

Nsyl :  GATCCAACTGGTGCAACTATGATAAGAACTTATCTTACATTTTAG : 1575
Nt-1 :  GATCCAACTGGTGCAACTATGATAAGAACTTATCTTACATTTTAG : 1575
```

Coding sequence comparison between *Ntε-LCY2* (Nt-2) and *Ntom-LCY* (Ntom)

```
Ntom : ATGGAGTGTATTGGAGCTCGAAATTTTGCTACAATGGCGGTTTTTACGTGTCCGAGATTCAAATCATTAGGAAGAAGGAGAATTATGCCA :  90
Nt-2 : ATGGAGTGTATTGGAGCTCGAAATTTTGCTACAATGGCGGTTTTTACGTGTCCGAGATTCAAATCATTAGGAAGAAGGAGAATTATGCCA :  90

Ntom : AGAAAAAAGCAACCATTTTGGCCTATACATATGCAAGTGAAGTGTAGTGGAAATGAGAGTTGTGTAGTAGTTAAAGAAGATTTTGCTGAT : 180
Nt-2 : AGAAAAAAGCAACCAATTTGGCCTATACATATGCAAGTGAAGTGTAGTGGAAATGAGAGTTGTGTAGTAGTTAAAGAAGATTTTGCCGAT : 180

Ntom : GAAGAGGATTATATAAAAGCTGGTGGTTCAGAACTTGTTTTTGTTCAAATGCAGCAGAATAAAGACATGGATCTGCAGTCTAAGCTTTCT : 270
Nt-2 : GAAGAGGATTATATAAAAGCTGGTGGTTCAGAACTTGTTTTTGTTCAAATGCAGCAGAATAAAGACATGGATCTGCAGTCTAAGCTTTCT : 270

Ntom : GATAAGTTGCGACAAATATCATCAACTGGACAAACTATACTGGATTTGGTGGTCATAGGTTGTGGTCCTGCTGGTCTTGCTCTTGCCGCG : 360
Nt-2 : GATAAGTTGCGACAAATATCATCAGCTGGACAAACTATACTGGATTTGGTGGTCATAGGCTGTGGTCCTGCTGGTCTTGCTCTTGCTGCG : 360

Ntom : GAGTCTGCTAAACTCGGATTGAACGTTGGGCTCGTTGGTCCTGATCTTCCTTTCACAAATAACTATGGTGTTTGGGAGGATGAGTTCAAA : 450
Nt-2 : GAGTCTGCTAAACTCGGATTGAACGTTGGGCTCGTTGGTCCTGATCTTCCTTTCACAAATAACTATGGTGTTTGGGAGGATGAGTTCAAA : 450
```

```
Ntom : GATCTTGGACTTCAAGCGTGCATTGAACATGTATGGAGGGATACCATTGTATATCTTGACGATGCCGATCCAATTCTTATCGGTCGTGCT :  540
Nt-2 : GATCTTGGGCTTCAAGCGTGCATTGAACATGTTTGGAGGGATACCATAGTATATCTTGACGATGCCGATCCAATTCTTATCGGTCGTGCT :  540

Ntom : TATGGAAGAGTTAGTCGCCATTTACTGCACGAGGAGTTACTCAAAAGGTGTGTGGAGGCAGGTGTTTTATATCTTAACTCGAAAGTGGAT :  630
Nt-2 : TATGGAAGAGTTAGTCGCCATTTACTGCACGAGGAGTTACTCAAAAGGTGTGTGGAGGCAGGTGTTTTATATCTTAACTCGAAAGTGGAT :  630

Ntom : AGGATCGTTGAGTCCACAAGTGGCCACAGTCTTGTAGAGTGCGAGGGCGACATTGTCATTCCTTGCAGGTTTGTCACTGTTGCATCTGGA :  720
Nt-2 : AGGATCGTTGAGTCCACAAGTGGCCACAGTCTTGTAGAGTGCGAGGGCGACATTGTCATTCCTTGCAGGTTTGTCACTGTTGCATCTGGT :  720

Ntom : GCCGCCTCAGGGAAATTCTTGCAGTATGAGTTGGGAGGTCCTCGGGTTTCTGTTCAAACAGCTTATGGAGTGGAAGTTGAGGTCGATAAC :  810
Nt-2 : GCTGCCTCAGGGAAATTCTTGCAGTATGAGTTGGGAGGTCCTCGGGTTTCTGTTCAAACAGCTTATGGAGTGGAAGTTGAGGTCGATAAC :  810

Ntom : AATCCGTATGATCCAAGTCTGATGGTTTTCATGGATTATAGAGACTATGTCAGACACGACGCTCAATCTTTAGAAGCTAAATATCCAACA :  900
Nt-2 : AATCCGTATGATCCAAGCCTGATGGTTTTCATGGATTATAGAGACTATGTCAGACACGACGCTCAATCTTTAGAAGCTAAATATCCAACA :  900

Ntom : TTTCTTTATGCCATGCCCATGACTAAAACAAGAGTCTTTTTCGAGGAAACTTGTTTGGCTTCAAAAGATGCAATGCCATTTGATTTGTTA :  990
Nt-2 : TTTCTTTATGCCATGCCCATGACTAAAACAAGAGTCTTTTTCGAGGAAACTTGTTTGGCTTCAAAAGATGCAATGCCATTTGATTTGTTA :  990

Ntom : AAGAAAAAGCTGATGTTACGATTGAACACACTGGGTGTAAGAATTAAGCAAATCTACGAGGAGGAATGGTCTTACATACCAGTTGGTGGA : 1080
Nt-2 : AAGAAAAAACTGATGTTACGATTGAACACACTGGGTGTAAGAATTAAGCAAATCTACGAGGAGGAATGGTCATACATACCAGTTGGTGGA : 1080

Ntom : TCGTTGCCAAATACAGAGCAGAAAACACTTGCGTTTGGCGCTGCTGCTAGCATGGTTCATCCAGCTACAGGTTATTCAGTTGTCAGATCA : 1170
Nt-2 : TCTTTACCAAATACCGAGCAAAAAACACTTGCATTTGGTGCTGCTGCTAGCATGGTTCATCCAGCTACAGGTTATTCAGTTGTCAGATCA : 1170

Ntom : CTGTCCGAGGCGCCAAAATGCGCCTCAGTACTTGCTAATATTTTAAGACAAAATCATGTCAAGAACATGCTTACTAGTTCAAGTACCACA : 1260
Nt-2 : CTGTCCGAGGCACCAAAATGCGCCTCCGTACTTGCTAATATTTTACGACAAAATCATGTCAAGAACATGCTAACCAGTTCAAGTACCACA : 1260

Ntom : AGTATCTCAACTCAAGCTTGGAACACCCTTTGGCCACAAGAACGAAAAAGGCAAAGATCGTTTTTCCTATTTGGCTTGGCACTCATATTG : 1350
Nt-2 : AGTATCTCAACTCAAGCTTGGAACACCCTTTGGCCACAAGAACGAAAAAGGCAACGATCGTTTTTCCTATTTGGATTGGCACTCATATTG : 1350

Ntom : CAGTTGGATATTGAGGGGATTAGGTCATTTTTCCGCGCATTCTTCCTTGTGCCAAAATGGATGTGGCAAGGATTTCTTGGCTCTAGTCTT : 1440
Nt-2 : CAGTTGGATATTGAGGGGATTAGGTCATTTTTCCGCGCATTCTTCCGTGTGCCAAAATGGATGTGGCAAGGATTTCTTGGCTCTAGTCTT : 1440

Ntom : TCATCAGCAGACCTCATGTTATTTGCCTTCTACATGTTTATTATTGCACCAAATGACATGAGAAAAGGCCTAATCAGACATCTGTTATCT : 1530
Nt-2 : TCATCAGCAGACCTCATGTTATTTGCCTTCTACATGTTTATTATTGCACCAAATGACATGAGAAAAGGCCTAATCAGACATTTGTTATCT : 1530

Ntom : GATCCAACTGGTGCAACCATGATAAGAACTTATCTTACATTTTAG : 1575
Nt-2 : GATCCAACTGGTGCAACCATGATAAGAACTTATCTTACATTTTAG : 1575
```
